# Risk-taking to obtain reward: sex differences and associations with emotional and depressive symptoms in a nationally representative cohort of UK adolescents

**DOI:** 10.1017/S0033291720005000

**Published:** 2022-10

**Authors:** Gemma Lewis, Ramya Srinivasan, Jonathan Roiser, Sarah-Jayne Blakemore, Eirini Flouri, Glyn Lewis

**Affiliations:** 1 Division of Psychiatry, Faculty of Brain Sciences, University College London, London, UK; 2 Division of Psychology & Language Sciences, Institute of Cognitive Neuroscience, University College London, London, UK; 3 Department of Psychology, Division of Psychology & Language Sciences, University of Cambridge; and Institute of Cognitive Neuroscience, University College London, London, UK; 4 IOE – Psychology & Human Development, UCL Institute of Education, London, UK

**Keywords:** Depression, reward, cambridge gambling task, epidemiology, adolescent

## Abstract

**Background:**

Cognitive mechanisms that characterize or precede depressive symptoms are poorly understood. We investigated cross-sectional and longitudinal associations between risk taking to obtain reward and adolescent depressive symptoms in a large prospective cohort, using the Cambridge Gambling Task (CGT). We also explored sex differences.

**Methods:**

The Millennium Cohort Study (MCS) is an ongoing UK study, following the lives of 19 000 individuals born 2000/02. The CGT was completed at ages 11 (*n* = 12 355) and 14 (*n* = 10 578). Our main exposure was the proportion of points gambled, when the odds of winning were above chance (risk-taking to obtain reward). Outcomes were emotional symptoms (Strengths and Difficulties Questionnaire, SDQ) at age 11 and depressive symptoms (short Mood and Feelings Questionnaire, sMFQ) at age 14. We calculated cross-sectional and longitudinal associations, using linear regressions.

**Results:**

In univariable models, there was evidence of cross-sectional associations between risk-taking and SDQ/sMFQ scores, but these associations disappeared after we adjusted for sex. Longitudinally, there was weak evidence of an association between risk-taking and depressive symptoms in females only [a 20-point increase in risk-taking at age 11 was associated with a reduction of 0.31 sMFQ points at age 14 (95% CI −0.60 to −0.02)]. At both time-points, females were less risk-taking than males.

**Conclusions:**

We found no convincing evidence of a relationship between risk-taking to obtain reward and depressive symptoms. There were large sex differences in risk-taking, but these do not appear to contribute to the female preponderance of depressive symptoms in adolescence.

## Introduction

Depression is a major contributor to the global burden of disease and by 2030 is predicted to be the leading cause of disability in high-income countries (Mathers & Loncar, 2006). Depression often begins during adolescence and adolescence is important for primary prevention (Thapar, Collishaw, Pine, & Thapar, 2012). Around the age of 12–13, the incidence of depression increases sharply in females and, by adulthood, depression affects twice as many women as men (Thapar et al., 2012). The prevalence of depression has risen among young women in the past two decades (Mcmanus, Bebbington, Jenkins, & Brugha, 2016) and a better understanding of the causes of the gender difference in depressive symptoms would be informative for prevention.

Abnormalities in cognitive functioning are a major source of the disability associated with depressive illness and may be a cause of depression, rather than just a consequence (Roiser & Sahakian, 2013). One theory of depression proposes reduced connectivity between neural circuits for executive control (such as the prefrontal cortex) and neural circuits that respond to rewarding or punishing emotional information (such as the ventral striatum) (Eshel & Roiser, 2010). The precise mechanisms are poorly understood but may involve disrupted translation of reward sensitivity into reward-seeking behaviours such as risk-taking. According to this theory, reduced risk-taking and reward-seeking are potential risk factors for depression.

Risk-taking can be assessed using self-administered scales such as the Arnett scale (Arnett, 1994), where respondents agree or disagree with statements about risk-taking. However, self-report measures are prone to recall and social desirability biases, which can reduce validity. In contrast, behavioural tasks such as the Cambridge Gambling Task (CGT) or Iowa Gambling Task (IGT) ask participants to place bets, and decision making in risk-taking and reward-seeking contexts can be more objectively evaluated. A recent systematic review identified five studies of associations between the IGT and depression in adults (de Siqueira et al., 2018). Findings were inconsistent but there was some evidence that adults with depression performed worse on the task overall (compared to adults without depression). As far as we know, associations between the IGT and depression have never been examined in adolescents.

The CGT assesses decision making and risk-taking to obtain reward. In the CGT, participants guess the location of a hidden token, and then gamble a proportion of their points on their decision. If people with depression are less sensitive to rewards (and/or more sensitive to punishments), one would expect a smaller proportion of points to be gambled in the CGT, even when the odds of winning are high. A few studies have investigated associations between the CGT and depression in adults and found, for example, that people with depression are less likely (than healthy controls) to place high bets when reward probabilities are high (Clark et al., 2011; Halahakoon et al., 2020). Several studies have investigated associations between the CGT and depression in adolescents, but produced mixed findings. In a small case–control study of 15-year-olds, no differences were observed (Kyte, Goodyer, & Sahakian, 2005). In another small case–control study of adolescents with a family history of depression, cases were less risk-taking than controls but there was no difference on risk adjustment (Mannie, Williams, Browning, & Cowen, 2014). There have been two cohort studies, one of males aged 10–11 (Forbes, Shaw, & Dahl, 2007) and one of 10–18-year-olds with depressed parents (Rawal, Collishaw, Thapar, & Rice, 2013). In both studies, placing lower stakes when the odds of winning were high was associated with increased depressive symptoms 1 year later.

It is possible that cognitive risk factors for adolescent depression are more common in females than males and this contributes to the higher incidence of depression in females. Adolescent males are more risk-taking than females (Charness & Gneezy, 2012); however, sex differences have not been investigated on gambling tasks such as the CGT. Three adult studies of the CGT have found that men and women do not differ in the overall amount bet, but women exhibit lower risk-adjustment (Deakin, Aitken, Robbins, & Sahakian, 2004; van den Bos, Homberg, & de Visser, 2013; van den Bos, Taris, Scheppink, de Haan, & Verster, 2014).

Existing studies of the CGT and depression in adolescents are small (*n* < 200) and may have lacked statistical power to detect small effects (Button et al., 2013). They also use unrepresentative samples and might have been affected by selection bias. The two cohort studies used short follow-up periods (1 year) and one only assessed males up to the age of 12 (Forbes et al., 2007). Depression is rare in males until later in adolescence, so this study might have lacked statistical power to detect valid associations. The other cohort study contained very few males with depression, so had limited statistical power to investigate sex differences (Rawal et al., 2013).

We used a large nationally representative UK birth cohort to investigate cross-sectional and longitudinal associations between risk-taking to obtain reward on the CGT and adolescent depressive symptoms. We also examined sex differences in performance on the task and compared cross-sectional and longitudinal associations in females and males.

## Methods

### Sample

The Millennium Cohort Study (MCS) is an ongoing nationally representative study of 18 552 families and 18 818 children born in the UK between 2000 and 2002 (Joshi & Fitzsimons, 2016). The target population, from which every child had a chance of being selected, was defined as those born within eligible dates: between September 2000 and August 2001 in England and Wales and between November 2000 and January 2002 in Scotland and Northern Ireland; alive and living in the UK at age 9 months and eligible to receive Child Benefit at that age. Probability (or random) methods of selection were used. The primary sampling unit was geographical area defined by electoral wards and the population was stratified by UK country, ethnic background and social deprivation. More socially deprived and ethnically diverse areas were oversampled to increase representation. Six waves of data have been collected: when children were aged around 9 months (MCS1), 3 (MCS2), 5 (MCS3), 7 (MCS4), 11 (MCS5) and 14 years (MCS6). Further details are available on the study website: https://cls.ucl.ac.uk/cls-studies/millennium-cohort-study/.

We used data from ages 11 and 14 (when the CGT was available), excluding 246 twins and 10 triplets so that data were independent. Flow of participants through the study is shown in online Supplementary Fig. S1. All families gave written informed consent. Data were obtained from the UK Data Archive. Ethics approval for the MCS was obtained from the UK National Health Service Research Ethics Committee (ref: 11/YH/0203). The authors report no conflict of interests.

### Outcome

#### Adolescent depressive symptoms

Our primary outcome was the short Moods and Feelings Questionnaire (sMFQ), completed at 14 years of age (only). The sMFQ is a 13-item self-report measure of DSM-IV depressive symptoms in the past 2 weeks, ranging from 0 to 26, with higher scores indicating more severe symptoms. The sMFQ is a widely used measure of adolescent depressive symptoms, with excellent psychometric properties including high sensitivity and specificity (Thapar & McGuffin, 1998). Reliability was high (Cronbach's *α* 0.97). A score of 12 or above indicates potential clinical significance (Thapar & McGuffin, 1998). We used the sMFQ total score as our main outcome and report the proportion exceeding the cut-off as a descriptive.

#### Child emotional symptoms

The Strengths and Difficulties Questionnaire (SDQ) has five sub-scales assessing emotional symptoms, conduct problems, hyperactivity, peer problems and prosocial behaviour. The SDQ was completed by the parent (mostly mothers) at multiple waves, when children were aged 3–14. We used the five-item emotional symptoms sub-scale as the outcome in our age 11 cross-sectional analyses (only), as there was no depression measure. Analyses at age 14 used the sMFQ as the outcome.

#### Exposure

The CGT was completed on a computer at ages 11 and 14. Participants were shown 10 red or blue boxes at the top of the screen. At the bottom of the screen were two more boxes, labelled either red or blue. Participants were told that a yellow token was hidden underneath one of the boxes at the top of the screen. They had to guess whether the token was hidden under a red or blue box, and indicate their choice by selecting a box at the bottom of the screen (online Supplementary Fig. S2). The task was in five stages. Stage one was a ‘decision-only’ practice stage with four trials: on each trial, the task was to indicate whether the token was hidden under a red or blue box. In the first trial, the interviewer demonstrated, and in the remaining three trials, the participant practised.

After the decision-only stage, the gambling stages (stages two to five) began. Participants were given 100 points and told that their aim was to get as many points as possible before the task ended. After selecting the colour of the box hiding the token, participants were asked to bet a percentage of their points on how confident they were. The percentages were individually flashed onto the screen in 5 s intervals (5, 25, 50, 75, 95%). The participant selected a percentage and the location of the token was revealed. The value of the bet was either added to (if correct) or subtracted from (if incorrect) the point total (Rawal et al., 2013).

In the first two gambling stages (stages two and three), bets were presented in ascending order (5, 25, 50, 75, 95%). Stage 2 was a practice, with four trials. The interviewer demonstrated the first and the participant practised the remaining three. The ascending stage then began, with two blocks of nine trials. In the last two gambling stages (stages four and five), bets were presented in descending order (95, 75, 50, 25, 5%; first a practice stage of four trials, then an assessed stage with two blocks of nine trials).

Two outcome variables were derived from the CGT, consistent with previous studies (Murphy et al., 2001) (full details on how the variables were derived can be found here: https://cls.ucl.ac.uk/wpcontent/uploads/2017/07/mcs5_cantab_assessments_data_note.pdf).

1. Risk-taking to obtain reward: The mean percentage of the current points total that participants choose to gamble, on trials when they selected the most likely outcome (e.g. trials when they selected a red box, when there were more red than blue boxes). The risk-taking variable is therefore the proportion bet (restricted to trials where participants choose the more probable colour), irrespective of the odds of winning (i.e. whether on 6:4, 7:3, 8:2 or 9:1 trials). Percentages range from 5% to 95% so the risk-taking measure ranges from 0.05 to 0.95. We converted these proportions to percentages (ranging from 5% to 95%).
2. Risk adjustment: Participants should bet a higher proportion of their points on a certain colour when more of the boxes are that colour. For example, participants should bet more on a red box when the ratio of boxes is 9:1 (red:blue) than when it is 6:4 (red:blue). Risk adjustment measures the extent to which, on trials where a larger proportion of boxes are a certain colour, participants bet a higher proportion of their points. Higher risk adjustment scores represent a higher proportion of points bet as ratio increases. Risk adjustment is calculated using the following formula:

(2*a* + *b* – *c* – 2*d*)/*e*,

*a* = mean proportion bet where the chosen colour ratio is 9:1;

*b* = mean proportion bet where the chosen colour ratio is 8:2;

*c* = mean proportion bet where the chosen colour ratio is 7:3;

*d* = mean proportion bet where the chosen colour ratio is 6:4;

*e* = mean proportion risked over all trials.

#### Potential confounders

We identified variables that might be alternative explanations of the association between exposure and outcome, but not on the causal pathway between them. We included family income, maternal education, child age at the time of the exposure, ethnic background, stage of pubertal development, child cognitive ability (as a proxy for intelligence quotient), parent depressive symptoms and, in longitudinal analyses, children's baseline emotional symptoms and behavioural problems. Details on how these were measured are included in the Supplementary material.

## Statistical analyses

All analyses were conducted in Stata version 14, weighted to account for the MCS sampling design and representativeness. We applied the sampling weights before analyses were run, so that standard errors were adjusted to account for possible effects of stratification and clustering. We present baseline characteristics in the sample overall, and according to exposure status using *t* tests or χ^2^ tests for continuous or categorical characteristics, respectively.

### Sex differences in risk-taking and risk adjustment

We investigated sex differences in risk-taking and risk adjustment at ages 11 and 14 using univariable linear regression models. We also present these linear regression models after adjusting for the variables we considered as confounders in other analyses. In the sample overall, and according to sex, we calculated the correlation between risk-taking at ages 11 and 14, and did the same for risk adjustment.

### Cross-sectional associations at 11 and 14 years of age

First, we tested a univariable linear regression model with risk-taking (continuous exposure) and emotional symptoms (continuous outcome) at 11 years of age. Next, we adjusted for potential confounders assessed at the time of assessment (age 11) or as close as possible to it. Next, we adjusted for sex. We also calculated an interaction between sex and risk-taking, to test whether the association varied by sex. The same analyses were conducted at age 14, with depressive symptoms as the outcome.

### Longitudinal associations

First, we tested a univariable linear regression model with risk-taking (continuous exposure) at age 11 and depressive symptoms (continuous outcome) at age 14. Next, we adjusted for potential confounders at baseline, and then for sex. We calculated an interaction between sex and risk-taking. These analyses were repeated with the age 11 risk adjustment variable as the exposure.

### Missing data

Our primary analyses for each aim used a sample with complete data on all variables required for that aim (cross-sectional or longitudinal). First, we investigated differences between the samples with complete and missing data. In cross-sectional analyses, we used a population weight to adjust the sample to be representative of the population. This would also account for differences between complete case samples and the target population that were due to missing data. In the longitudinal analysis, we used a non-response weight which accounted for potential attrition biases and adjusted the sample to be representative of the population.

As a sensitivity analysis, we replaced missing data with multiple imputation using chained equations (MICE) (Sterne et al., 2009). We assumed missing data were dependent on observed data (‘missing at random’) and imputed 50 datasets. To predict missing data, we used all variables in analysis models and auxiliary variables including SDQ measures at all time-points. For each analysis (cross-sectional or longitudinal), we started with a sample that had complete data on the exposure, and replaced the missing data in confounders and outcome. MICE allows for uncertainty about the true values of missing data, by creating multiple plausible imputed datasets and combining the results from each. Missing values are replaced by imputed values sampled from their predictive distribution based on the observed data. Standard methods of analysis (e.g. regression models) are then used and results are averaged to give overall estimations, with standard errors calculated using Rubin's rules (Sterne et al., 2009).

## Results

### Descriptive statistics

Complete data on exposure, outcome and confounders were available for 10 396 adolescents at age 11 (mean age 11 years, s.d. 0.48; 50% female; online Supplementary Fig. S1). At age 14, we had complete data on 8628 adolescents (mean age 14 years, s.d. 0.45; 50% female; online Supplementary Fig. S1). For longitudinal analyses, complete data were available for 8418 adolescents (online Supplementary Fig. S1). Participants who dropped out of the study by age 14 had higher risk-taking, parental depression and SDQ scores and tended to be from parents with lower incomes and educational qualifications. They were also more likely to be male and had lower verbal reasoning scores (online Supplementary Table S1).

Table 1 shows the characteristics of the sample overall, and according to risk-taking scores (using a median split of the risk-taking variable, to illustrate differences between those with higher and lower risk-taking, to identify potential confounders). Children who were less risk-taking at age 11 were more likely to be female, less likely to be an ethnic minority and had parents with lower education and income (Table 1). Less risk-taking children had lower parental depressive symptoms, lower SDQ total scores at age 11 and a higher mean verbal reasoning score (Table 1). There was no evidence of an association between risk-taking and pubertal status in females or males at age 11 (Table 1). At age 14, there was a similar pattern, but no differences for parental depressive symptoms (Table 2). There was no evidence of an association between pubertal status and risk-taking at age 15 in females. A larger proportion of males in the lowest pubertal status category were in the high-risk-taking group (compared with the low-risk-taking group).

**Table 1.** Characteristics of those with complete data at 11 years of age in the sample overall and according to reward-seeking split at the median (*n* = 10 396)

Characteristic	Sample overall	Risk-taking^a^		*p*^b^
		Low (*n* = 5436)	High (*n* = 4960)	
Mean parent depressive symptoms score (s.d.), range 0–24	3.80 (6.61)	3.72 (9.07)	3.91 (7.70)	0.069
Mean verbal reasoning score (s.d.), range 20–80	59.52 (25.21)	59.78 (20.06)	59.23 (22.56)	0.022
Mean SDQ total difficulties score (s.d.), range 0–36	7.26 (9.01)	6.77 (8.16)	7.81 (8.13)	<0.0001
Lowest income quintile	1754 (11%)	817 (10%)	937 (13%)	<0.0001
Compulsory maternal education only	4173 (44%)	2307 (46%)	1866 (42%)	<0.0001
Ethnic minority	1290 (9%)	560 (8%)	730 (10%)	<0.0001
Female	5159 (50%)	3300 (61%)	1859 (38%)	<0.0001
In female sub-sample (*n* = 5159): stage of breast development^c^				
Not yet started	987 (19%)	602 (19%)	385 (19%)	
Barely started	1644 (32%)	1059 (32%)	585 (32%)	
Definitely started	2527 (49%)	1638 (49%)	889 (48%)	0.65
In male sub-sample (*n* = 5237): stage of facial hair development				
Not yet started	4778 (91%)	1941 (91%)	2837 (92%)	
Barely started	356 (7%)	147 (7%)	209 (7%)	
Definitely started	103 (2%)	48 (2%)	55 (1%)	0.47

Data are mean (s.d.) or *n* (%).

*N*s are unweighted; means, standard deviations and percentages are weighted to be representative of the sample overall.

a Split at the median value for risk-taking, 55.

b *p* Value for the comparison between low- and high-risk-taking obtained from *t* tests for continuous characteristics and χ^2^ tests for categorical characteristics.

^c^Data on puberty missing for one female adolescent adolescent.

**Table 2.** Characteristics of the sample with complete data at 14 years of age, according to risk-taking split at the median (*n* = 8628)

Characteristic	Sample overall	Risk-taking^a^		*p*^b^
		Low (*n* = 4515)	High (*n* = 4113)	
Mean parent depressive symptoms score (s.d.), range 0–24	4.08 (5.67)	4.07 (5.49)	4.09 (4.82)	0.80
Mean adolescent vocabulary score (s.d.), range 0–19	7.39 (4.72)	7.38 (4.34)	7.01 (4.17)	<0.0001
Lowest income quintile	1056 (9%)	477 (8%)	579 (10%)	<0.0001
Compulsory maternal education	3672 (46%)	1976 (47%)	1696 (45%)	0.018
Ethnic minority	1138 (9%)	531 (9%)	607 (10%)	<0.0001
Female	4354 (50%)	2724 (61%)	1630 (38%)	<0.0001
In female sub-sample (*n* = 4354): stage of breast development^c^				
Not yet started	49 (1%)	26 (1%)	23 (1%)	
Barely started	897 (19%)	560 (19%)	337 (19%)	
Definitely started	2962 (69%)	1865 (70%)	1097 (68%)	
Seems completed	445 (11%)	273 (10%)	172 (11%)	0.50
In male sub-sample (*n* = 4274): stage of facial hair development				
Not yet started	851 (21%)	321 (19%)	530 (22%)	
Barely started	1865 (44%)	799 (44%)	1066 (43%)	
Definitely started	1474 (34%)	640 (35%)	834 (33%)	
Seems completed	84 (2%)	31 (2%)	53 (2%)	0.029

Data are mean (s.d.) or *n* (%)

*N*s are unweighted; means, standard deviations and percentages are weighted to be representative of the sample overall.

a Split at the median value for risk-taking, 52.

b *p* Value for the comparison between low- and high-risk-taking obtained from *t* tests for continuous characteristics and χ^2^ tests for categorical characteristics.

^c^Data on puberty missing for one female adolescent.

The mean SDQ emotional symptoms score at 11 years of age was 1.82 (s.d.) in the sample overall, 1.90 (s.d.) in females and 1.73 (s.d.) in males. The mean sMFQ depressive symptoms score at 14 years of age was 5.62 (s.d. 8.21) in the sample overall, 7.20 (s.d. 9.52) in females and 4.01 (s.d. 5.51) in males. On the sMFQ at age 14, 1567 (18%) adolescents met the recommended criteria for symptoms of clinical significance, 1135 (72%) females and 432 (28%) males.

### Sex differences in risk-taking and risk adjustment

In the sample overall, the mean risk-taking score at 11 years of age was 52.60 (s.d. 16.92). By 14 years of age, this had decreased slightly to 51.35% (s.d. 14.67; mean difference 1.00, 95% CI 0.63–1.37, *p* < 0.0001). The correlation between risk-taking scores at ages 11 and 14 was *r* = 0.33 (*p* < 0.0001).

At each time-point, males were more risk-taking than females (Tables 1 and 2). At 11 years of age, the mean risk-taking score for males was 57.6% (s.d. 15.71) and for females 48.3% (s.d. 16.75). The unadjusted mean difference in risk-taking between males and females, from weighted linear regression, was 9.31 percentage points (95% CI 8.59–10.03, *p* < 0.0001). After adjustments, the mean difference was 9.39 (95% CI 8.66–10.10, *p* < 0.0001). At 14 years of age, the mean risk-taking score had decreased slightly in males (55.59%, s.d. 14.33%) and remained similar in females (48.10%, s.d. 14.36%); the mean difference from weighted linear regression was 7.52 percentage points (95% CI 6.87–8.17, *p* < 0.0001) before adjustments and after, 7.57 (95% CI 6.92–8.23, *p* < 0.0001). The correlation between risk-taking at the two ages was similar in males (*r* = 0.28, *p* < 0.0001) and females (*r* = 0.28, *p* < 0.0001).

In the sample overall, the mean risk adjustment score at 11 years of age was 0.67 (s.d. 1.03). By 14 years of age, this had increased to 1.04 (s.d. 0.98). The correlation between risk adjustment at ages 11 and 14 was *r* = 0.26 (*p* < 0.0001).

At 11 years of age, there was no evidence of a sex difference in risk adjustment. The mean risk adjustment score for males was 0.68 (s.d. 1.03) and for females 0.66 (s.d. 1.04); mean difference 0.03 (95% CI −0.01 to −0.07, *p* = 0.20). At 14 years of age, the mean risk adjustment score had increased in females and males but to a larger extent in males (mean, males: 1.15, s.d. 0.98; mean, females: 0.96, s.d. 0.97); mean difference from weighted linear regression before (0.16, 95% CI 0.11–0.21, *p* < 0.0001) and after (0.15, 95% CI 0.10–0.20, *p* < 0.0001) adjustments. The correlation between risk adjustment across time-points was similar in males (*r* = 0.27, *p* < 0.0001) and females (*r* = 0.25, *p* < 0.0001).

### Cross-sectional associations

Mean SDQ and sMFQ scores according to risk-taking and risk adjustment are presented in online Supplementary Tables S2–S4.

Cross-sectional associations between risk-taking and emotional symptoms at 11 years of age are presented in Table 3. In a univariable model, we found no evidence of an association (−0.03, 95% CI −0.08 to 0.03, *p* = 0.314). After adjustments, there was stronger evidence of a negative association (−0.07, 95% CI −0.12 to −0.01, *p* = 0.015). This association attenuated after accounting for sex (−0.04, 95% CI −0.09 to 0.01, *p* = 0.122). When associations were examined separately in males and females (i.e. the potentially confounding role of sex is removed), there was no evidence of an association between risk-taking and emotional symptoms, indicating confounding by sex. There was no evidence of an interaction between risk-taking and sex (*p* = 0.99).

**Table 3.** Unstandardized regression coefficients, and 95% confidence intervals, for the change in SDQ scores (continuous outcome) per 20-point increase in risk-taking (continuous exposure) at age 11, in the complete-case sample (*n* = 10 396)

Sample overall	Coefficient (95% CI)	*p*
Model 1a: Univariable (*n* = 10 396)	−0.03 (−0.08 to 0.03)	0.314
Model 1b: Model 1a adjusted^a^	−0.07 (−0.12 to −0.01)	0.015
Model 1c: Model 1b adjusted for sex	−0.04 (−0.09 to 0.01)	0.122
Sub-group analyses by sex	Coefficient (95% CI)	*p*
Model 2a: Univariable, females (*n* = 5159)	0.01 (−0.07 to 0.09)	0.752
Model 2b: Model 2a adjusted^b^	−0.04 (−0.11 to 0.03)	0.299
Model 3a: Univariable, males (*n* = 5237)	0.00 (−0.07 to 0.08)	0.987
Model 3b: Model 3a adjusted^b^	−0.04 (−0.11 to 0.03)	0.217

a Adjusted for confounders measured at or as close as possible to baseline (age 11): family income, maternal education, child age, child ethnicity, child verbal reasoning score, main carer depressive symptoms.

b Adjusted for the above confounders and, in addition, stage of breast development in females and facial hair development in males at age 11.

We tested for a non-linear association between risk-taking and emotional symptoms with a quadratic term in the fully adjusted model. There was no evidence of deviation from linearity (*p* value for quadratic term 0.18).

When analyses were repeated with risk adjustment as the exposure, there was no evidence of an association with emotional symptoms after adjustment for confounders (online Supplementary Table S5).

Cross-sectional associations between risk-taking and depressive symptoms at 14 years of age are presented in Table 4. In the univariable model, we found strong evidence of an association. For each 20-point increase in risk-taking, depressive symptoms decreased by 0.52 of an MFQ point (95% CI −0.71 to −0.33, *p* < 0.0001). This was hardly altered after adjusting for confounders (−0.54, 95% CI −0.73 to −0.35, *p* < 0.0001) but substantially reduced after accounting for sex (0.05, 95% CI −0.15 to 0.25, *p* = 0.637). In associations stratified by sex, there was no evidence of an association. There was no evidence of an interaction between risk-taking and sex (*p* = 0.97).

**Table 4.** Unstandardized regression coefficients, and 95% confidence intervals, for the change in sMFQ scores (continuous outcome) per 20-point increase in risk-taking (continuous exposure) at age 14, in the complete-case sample (*n* = 8628)

Sample overall	Coefficient (95% CI)	*p*
Model 1a: Univariable (*n* = 8628)	−0.52 (−0.71 to −0.33)	<0.0001
Model 1b: Model 1a adjusted^a^	−0.54 (−0.73 to −0.35)	<0.0001
Model 1c: Model 1b adjusted for sex	0.05 (−0.15 to 0.25)	0.637
Sub-group analyses by sex	Coefficient (95% CI)	P
Model 2a: Univariable, females (*n* = 4354)	0.08 (−0.29 to 0.44)	0.680
Model 2b: Model 2a adjusted^b^	0.02 (−0.34 to 0.38)	0.893
Model 3a: Univariable, males (*n* = 4274)	0.05 (−0.16 to 0.26)	0.612
Model 3b: Model 3a adjusted^b^	0.08 (−0.14 to 0.29)	0.472

a Adjusted for confounders measured at or as close as possible to the time of the exposure (age 14): family income, maternal education, child age, child ethnicity, child vocabulary score, main carer depressive symptoms.

b Adjusted for the above confounders and, in addition, stage of breast development in females or facial hair development in males at age 14.

When analyses were repeated with risk adjustment as the exposure, there was no evidence of an association with depressive symptoms after adjustment for confounders (online Supplementary Table S6).

### Longitudinal association

Longitudinal associations are presented in Table 5. In the univariable model, we found strong evidence that, for every 20-point increase in risk-taking at age 11, depressive symptoms decreased by 0.55 of an MFQ point at age 14 (95% CI −0.76 to −0.34, *p* < 0.0001). After adjusting for confounders, this association was very similar (−0.65, 95% CI −0.85 to −0.44, *p* < 0.0001), but reduced substantially after we adjusted for sex (−0.13, 95% CI −0.33 to 0.07, *p* = 0.196). In analyses stratified by sex, there was weak evidence of an association in females (−0.31, 95% CI −0.60 to −0.02, *p* = 0.037). There was no evidence of an association in males (0.11, 95% CI −0.11 to 0.34, *p* = 0.328). There was weak evidence of an interaction between risk-taking and sex (*p* = 0.015).

**Table 5.** Unstandardized regression coefficients, and 95% confidence intervals, for the change in sMFQ scores at age 14 (continuous outcome) per 20-point increase in risk-taking at age 11 (continuous outcome), in the complete-case sample (*n* = 8418)

Sample overall	Coefficient (95% CI)	*p*
Model 1a: Univariable (*n* = 8418)	−0.55 (−0.76 to −0.34)	<0.0001
Model 1b: Model 1a adjusted^a^	−0.65 (−0.85 to −0.44)	<0.0001
Model 1c: Model 1b adjusted for sex	−0.13 (−0.33 to 0.07)	0.196
Sub-group analyses by sex	Coefficient (95% CI)	*p*
Model 2a: Univariable, females (*n* = 4257)	−0.27 (−0.58 to 0.04)	0.083
Model 2b: Model 2a adjusted^b^	−0.31 (−0.60 to −0.02)	0.037
Model 3a: Univariable, males (*n* = 4161)	0.19 (−0.05 to 0.43)	0.121
Model 3b: Model 3a adjusted^b^	0.11 (−0.11 to 0.34)	0.328

a Adjusted for confounders measured at or as close as possible to the time of the exposure (age 11): family income, maternal education, main carer depressive symptoms, child age, child ethnicity, child verbal reasoning score and SDQ total difficulties score.

b Adjusted for the above confounders and, in addition, stage of breast development in females or facial hair development in males at age 11.

With risk adjustment as the exposure, there was no evidence of an association with depressive symptoms after adjustment for confounders (online Supplementary Table S7).

### Sensitivity analyses

A similar pattern of associations for risk-taking was observed cross-sectionally and longitudinally in imputed samples (online Supplementary Tables S8–S10). In the imputed data, the longitudinal association between risk-taking and depressive symptoms was weaker (−0.24, 95% CI −0.47 to −0.00, *p* = 0.047). Imputed results for risk adjustment are not presented because this variable showed less evidence of an association with mood in the complete-case analyses (available on request).

## Discussion

### Summary of findings

In a large, nationally representative UK cohort, we did not find convincing evidence that risk-taking to obtain reward, assessed with the CGT, was associated with depressive or emotional symptoms in adolescents. In cross-sectional investigations, any evidence of an association between reduced risk-taking to obtain reward and depressive or emotional symptoms disappeared after we accounted for sex. When the associations were run separately in males and females (removing sex as a confounder), there was no evidence of any association between risk-taking to obtain reward and emotional/depressive symptoms.

In our longitudinal (but not cross-sectional) investigations, there was weak evidence that females (but not males) who were less risk-taking at age 11 had more depressive symptoms at age 14. Statistical evidence for this association was weak and sub-group analyses and interactions can be unreliable, especially when there is no main effect (Rothman, Greenland, & Lash, 2013). In the larger multiply imputed sample, evidence for an interaction and an association in females was even weaker, reducing our confidence in this finding. Our findings are inconsistent with other cohort studies (Forbes et al., 2007; Rawal et al., 2013). However, our sample was larger and we adjusted for a wider range of confounders, so it is possible that prior findings are due to chance, selection bias or confounding.

Consistent with wider literature on risk-taking, females were less risk-taking than males at 11 and 14 years of age, and risk-taking did not alter much in either sex across these ages. Our results add to the existing literature by suggesting that sex differences in risk-taking are unlikely to be a cognitive mechanism underlying the emergence of the sex difference in depression during mid-adolescence.

Consistent with adult studies, we found a large sex difference in risk adjustment at age 14 but there was no evidence of this at age 11. Risk adjustment increased in both males and females between 11 and 14, but to a larger extent in males. This suggests that adult sex differences in risk-taking and reward-seeking might emerge between the ages 11 and 14. In contrast to adult studies of the CGT, we found that, in addition to increasing their bets more as odds increased, adolescent males bet a larger proportion of their points than females (on average across all ratios). One possibility is that prior adult studies lacked statistical power to detect this difference. An alternative possibility is that adolescent sex differences in risk-taking decline with age.

### Strengths and limitations

This is, to our knowledge, the largest study of risk-taking and reward-seeking and adolescent depressive symptoms. Our sample was representative of UK adolescents and cohort studies are less prone to biases than case–control designs. The 3-year follow-up was a strength and included mid-adolescence when depression incidence increases, particularly in females.

Our study has several limitations. Prior longitudinal studies found that associations between risk-taking and depressive symptoms were stronger when the likelihood of obtaining a reward was higher (Forbes et al., 2007; Rawal et al., 2013). We could not restrict our analyses to trials with more favourable odds because raw data were unavailable. However, risk adjustment measures the extent to which participants bet a higher proportion of their points as the probability of winning increases. If participants were placing a lower proportion of their bets at high probabilities, this would be reflected in a lower risk adjustment estimate and we observed no evidence of this.

The CGT conflates reward-seeking and avoidance of punishment (i.e. in this case, losing points). Reduced betting, even when odds of winning are high, might occur either because participants are less motivated by reward or because they want to avoid punishment. The CGT also conflates risk-taking and reward-seeking and is probably a better measure of the former. It may be that tasks which separate the cognitive mechanisms of risk-taking from reward-seeking are associated with depression.

In our cross-sectional investigation at age 11, we used the parent-reported emotional symptoms sub-scale of the SDQ as the outcome, since no depressive symptoms measure was available. The SDQ is a broader concept than depression and is parent- rather than adolescent-reported. Although adolescent reports of their depressive symptoms would have been preferable, depressive symptoms are relatively uncommon and difficult to reliably assess at age 11, so the broader emotional problems scale of the SDQ might better capture mood disturbances at this age. In longitudinal analyses, we adjusted for children's emotional and behavioural problems rather than depressive symptoms at baseline. Adjusting for a wider range of symptoms at this age is likely to be a better way of accounting for differences in future depression risk, although residual confounding by childhood depression is still possible. We assessed pubertal status using a single-item measure which, although strongly associated with depression in females (Joinson et al., 2012; Lewis et al., 2018), is likely to be a crude assessment of the complexities of pubertal development. Missing data are a limitation of all cohort studies. We used two methods to reduce the possibility of attrition and non-response bias and our findings were consistent across these approaches.

Finally, future studies could investigate the developmental trajectories of risk-taking and depressive symptoms, which are both likely to change during adolescence. We were unable to take this approach because at least three measurement points are recommended to calculate a trajectory and the CGT was only available at ages 11 and 14 (Whittaker & Khojasteh, 2017).

### Implications

We found some evidence, albeit weak, that lower risk-taking might precede depressive symptoms in adolescent females. We interpreted this finding with caution, but it would be worth investigating in future studies with sufficient power to test interactions between depression and sex. Although we had a large sample, we might still have been underpowered to detect an association in males. The effect size we observed in females was small and there were fewer males with exposure and outcome. However, we cannot rule out the possibility that reduced risk-taking is a risk factor for depressive symptoms in females but not in males. Potential mechanisms underlying this association in females likely involve a complex combination of biological, psychological and social factors. Sex hormones, which were unavailable in the present study, are one potential avenue for future research, along with social and psychological mechanisms.

Our lack of evidence for associations between risk-taking to obtain reward and depressive symptoms might have occurred for several reasons. Risk-taking to obtain reward, at least how it is assessed in the CGT, might not be related to depressive symptoms in children and adolescents. This is an important possibility, and one we cannot rule out. If so, it has implications for how we think about the aetiology, treatment and prevention of depressive symptoms in children and adolescents. There is strong evidence, albeit in adults, that positive information processing is reduced in depression whilst negative information processing is unaltered (Lewis et al., 2017). It is possible that, in our study, the association between depressive symptoms and reward-seeking was masked by the conflation of positive and negative processing. It is also possible that more self-relevant or emotional information is important for emotional and depressive symptoms. In adult experimental psychopharmacology studies, antidepressants were found to modify recall of self-relevant personality descriptors and recognition of emotional facial expressions (Harmer, Duman, & Cowen, 2017). Adolescence is a time when peer influences become more important and social information might be more pertinent to depressive symptoms.

## Supplementary material

For supplementary material accompanying this paper visit https://doi.org/10.1017/S0033291720005000. click here to view supplementary material
