# Supplementary material for: Risk-taking to obtain reward: sex differences and associations with emotional and depressive symptoms in a nationally representative cohort of UK adolescents
Source: Psychol Med. 2021 Jan 12;52(13):2805–13. doi: 10.1017/S0033291720005000 (PMC9647510; doi:10.1017/S0033291720005000)
Supplement: Supplementary file 1 [file S0033291720005000sup.zip › S0033291720005000sup001.docx]

**Table of contents**

| **Content** | **Page** |
| --- | --- |
| Figure 1, sample flowchart | 2 |
| Figure 2, CGT example | 3 |
| Measurement of potential confounders | 4-5 |
| Post-hoc sample size calculation and minimum detectable effect | 5 |
| Table 1: Differences between samples with complete and missing data | 6 |
| Table 1: Mean SDQ scores according to risk taking and risk adjustment at age 11 | 7 |
| Table 2: Mean sMFQ scores according to risk taking and risk adjustment at age 11 | 7 |
| Table 3: Mean sMFQ scores at age 14 according to risk taking and risk adjustment at age 11 | 8 |
| Table 4: Risk adjustment as exposure, cross-sectional associations at age 11 | 9 |
| Table 5: Risk adjustment as exposure, cross-sectional associations at age 14 | 10 |
| Table 6: Risk adjustment as exposure, longitudinal associations | 11 |
| Table 7: Imputed model: cross-sectional risk taking association at age 11 | 12 |
| Table 8: Imputed model: cross-sectional risk taking association at age 14 | 13 |
| Table 9: Imputed model: Longitudinal risk taking association at age 11 | 14 |

**Supplementary Figures**

**<Insert supplementary figure 1 here>**

**<Insert supplementary figure 2 here>**

**Measurement of potential confounders**

Family income was total income adjusted for household size and composition, in quintiles. Maternal education ranged from 1 (none or compulsory education) to 6 (postgraduate), classified into compulsory and non-compulsory. Ethnic background was classified as white or ethnic minority due to small numbers. An adapted version of the pubertal development scale (PDS) (Petersen, Crockett, Richards, & Boxer, 1988) was used in MCS at ages 11 (parent reported) and 14 (adolescent reported). Consistent with prior studies (Flouri & Ioakeimidi, 2018; Kelly, Zilanawala, Sacker, Hiatt, & Viner, 2017), we selected certain indicators of pubertal development rather than using a sum score. We selected indicators of pubertal stage/status because this has been found to be more strongly associated with adolescent depressive symptoms than pubertal timing (Joinson et al., 2012; Lewis et al., 2018). We chose stage of breast development in females because it is more strongly associated with depression than age at menarche (Joinson et al., 2012) and also, for age at menarche, there is no easily obtained comparable measure for boys. Pubertal development was stage of breast development in females and facial hair development in males (parent report at age 11 and adolescent report at age 14). At age 11, children completed the Verbal Similarities test from the British Abilities Scale assessing verbal reasoning, fluency general knowledge and abstract and logical thinking. We used a derived variable, which was the total score age and ability adjusted (possible range of scores 20-80, higher scores indicating higher ability). At age 14 they completed a task that measured their understanding of word meanings and word knowledge (assessing vocabulary). The test contained 20 words, each followed by a multiple-choice list of 5 words from which the participant picked the word with the same meaning as the original word. Possible scores range from 0-20.A full description of each of the cognitive measures can be found here: <https://www.closer.ac.uk/wp-content/uploads/250820-Guide-to-cognitive-measures-in-five-British-birth-cohorts.pdf>. Parent depressive symptoms were measured using the Kessler 6-item psychological distress scale (K-6) (Kessler et al., 2002). In longitudinal analyses we adjusted for the parent reported SDQ total difficulties score, the sum of the scores on emotional, conduct, hyperactivity and peer problem scales (Goodman & Goodman, 2009).

**Post-hoc sample size and minimum detectable effect calculations**

We used the statistical software package G*Power to calculate minimum detectable effects given our sample size, assuming power of 99% and alpha of 5%.

For our investigation of the association between risk taking and depressive symptoms, our longitudinal analysis had the smallest sample size (n=8418). For this analysis, assuming 99% power and an alpha of 5%, we would be able to detect a standardised regression coefficient of 0.046.

For our investigations of sex differences in risk taking and risk adjustment, our cross-sectional investigation at age 14 had the smallest sample size (n=8628; 4354 girls and 4274 boys). For this analysis, assuming 99% power and an alpha of 5%, we would be able to detect a Cohen’s D effect size of 0.092.

Supplementary Table 1. Differences between samples with complete and missing data.

| Baseline characteristic (age 11) | Longitudinal analysis | | P value |
| --- | --- | --- | --- |
|  | Sample with complete data (8,418) | Sample with missing data (4,694) |  |
| Mean risk taking score (SD) | 52.3 (16.83) | 54.4 (16.90) | <.0001 |
| Mean risk adjustment score (SD) | 2.09 (1.41) | 1.82 (1.41) | <.0001 |
| Mean emotional symptoms score | 1.77 (1.93) | 2.08 (2.10) | <.0001 |
| Mean SDQ total difficulties score (SD) | 7.21 (5.49) | 8.84 (6.38) | <.0001 |
| Mean parent depressive symptoms score (SD) | 3.84 (4.23) | 4.70 (4.87) | <.0001 |
| Mean child cognitive ability score (SD) | 59.86 (9.30) | 56.40 (11.06) | <.0001 |
| Lowest income quintile | 1231 (14.62) | 1521 (32.40) | <.0001 |
| Compulsory maternal education only | 3647 (43.32) | 1043 (24.85) | <.0001 |
| Ethnic minority | 1081 (12.84) | 1191 (25.39) | <.0001 |
| Female | 4257 (50.57) | 2221 (47.32) | <.0001 |
| Outcome (age 14) |  |  |  |
| Mean depressive symptoms score | 5.59 (5.88) | 5.37 (5.85) | 0.12 |

**Means**

Supplementary Table 2. Mean (SD) SDQ scores at age 11, according to risk taking and risk adjustment in quintiles. Complete case sample (n=10,396).

| Risk taking quintiles | Mean (SD) | | | Risk adjustment quintiles | Mean (SD) | | |
| --- | --- | --- | --- | --- | --- | --- | --- |
|  | SDQ  overall | SDQ  females | SDQ  males |  | SDQ  overall | SDQ  females | SDQ  males |
| 1 (5-38) | 1.85 (1.93)  (n=2017) | 1.87 (1.91)  (n=1433) | 1.77 (1.98)  (n=584) | 1 (-.64 - -.09) | 2.04 (2.06)  (n=1997) | 2.10 (2.06)  (n=1034) | 1.97 (2.07)  (n=963) |
| 2 (39-47) | 1.82 (1.98)  (n=2199) | 1.83 (1.97)  (n=1216) | 1.80 (2.01)  (n=983) | 2 (-.08- -.35) | 1.87 (2.01)  (n=2028) | 2.03 (2.07)  (n=1019) | 1.72 (1.93)  (n=1009) |
| 3 (48-55) | 1.76 (1.94)  (n=1947) | 1.90 (2.01)  (n=978) | 1.63 (1.86)  (n=969) | 3 (.36-.78) | 1.79 (1.89)  (n=2065) | 1.83 (1.90)  (n=1029) | 1.74 (1.87)  (n=1036) |
| 4 (56-63,) | 1.80 (1.95)  (n=2197) | 2.01 (2.02)  (n=918) | 1.64 (1.89)  (n=1279) | 4 (.79-1.39) | 1.72 (1.93)  (n=2132) | 1.76 (1.89)  (n=1037) | 1.69 (1.96)  (n=1095) |
| 5 (64-95) | 1.86 (1.98)  (n=2036) | 1.95 (1.97)  (n=614) | 1.82 (1.98)  (n=1422) | 5 (1.40-6.00) | 1.68 (1.89)  (n=2174) | 1.80 (1.91)  (n=1040) | 1.57 (1.86)  (n=1134) |

Supplementary Table 3. Mean (SD) MFQ scores at age 14, according to risk taking and risk adjustment in quintiles. Complete case sample (n=8628).

| Risk taking quintiles | Mean (SD) | | | Risk adjustment quintiles | Mean (SD) | | |
| --- | --- | --- | --- | --- | --- | --- | --- |
|  | MFQ overall | MFQ females | MFQ males |  | MFQ overall | MFQ females | MFQ males |
| 1 (5-38) | 5.92  (n=1674) | 6.93  (n=1120) | 3.86  (n=554) | 1 (-.31-.021) | 5.79 (5.86)  (n=1608) | 7.12 (6.42)  (n=941) | 3.92 (4.31)  (n=667) |
| 2 (39-47) | 5.87  (n=1672) | 7.26  (n=969) | 3.96  (n=703) | 2 (-.22-.67) | 5.66 (5.94)  (n=1647) | 7.05 (6.54)  (n=803) | 4.05 (4.66)  (n=764) |
| 3 (48-55) | 5.70  (n=1903) | 7.27  (n=986) | 4.00  (n=917) | 3 (.68-1.14) | 5.43 (5.80)  (n=1712) | 6.89 (6.52)  (n=843) | 4.01 (4.58)  (n=869) |
| 4 (56-63,) | 5.35  (n=1652) | 7.06  (n=732) | 4.00  (n=920) | 4 (1.15-1.77) | 5.72 (6.04)  (n=1804) | 7.52 (6.80)  (n=872) | 4.04 (4.65)  (n=932) |
| 5 (64-95) | 4.99  (n=1727) | 6.80  (n=547) | 4.15  (n=1180) | 5 (1.78-5.10) | 5.26 (5.69)  (n=1857) | 6.83 (6.54)  (n=815) | 4.04 (4.57)  (n=1042) |

Supplementary Table 4. Mean (SD) MFQ scores at age 14, according to risk taking and risk adjustment quintiles, at age 11. Complete case sample (n=8418).

| Risk taking | Mean (SD) | | | Risk adjustment | Mean (SD) | | |
| --- | --- | --- | --- | --- | --- | --- | --- |
|  | MFQ overall | MFQ females | MFQ males |  | MFQ overall | MFQ females | MFQ males |
| 1 (5-37) | 6.37 (6.31)  (n=1652) | 7.18 (6.62)  (n=1192) | 4.27 (4.81)  (n=460) | 1 (-.64 - -.09) | 5.65 (5.81)  (n=1518) | 7.07 (6.51)  (n=812) | 4.01 (4.34)  (n=706) |
| 2 (38-49) | 5.87 (5.97)  (n=1815) | 7.19 (6.66)  (n=1072) | 4.15 (4.56)  (n=788) | 2 (-.08- -.35) | 5.55 (5.79)  (n=1625) | 7.00 (6.31)  (n=836) | 4.03 (4.73)  (n=789) |
| 3 (50-57) | 5.46 (5.85) (n=1591) | 6.97 (6.69)  (n=793) | 3.95 (4.40)  (n=798) | 3 (.36-.78) | 5.48 (6.01)  (n=1679) | 6.97 (6.86)  (n=857) | 3.93 (4.48)  (n=822) |
| 4 (58-67) | 5.32 (5.65)  (n=1784) | 7.07 (6.55)  (n=758) | 4.03 (4.46)  (n=1026) | 4 (.79-1.39) | 5.53 (5.83) (n=1754) | 6.74 (6.51)  (n=853) | 4.38 (4.84)  (n=905) |
| 5 (68-95) | 4.89 (5.51)  (n=1576) | 6.68 (6.54)  (n=487) | 4.09 (4.77)  (n=1089) | 5 (1.40-6.00) | 5.74 (5.97)  (n=1838) | 7.53 (6.76)  (n=899) | 4.03 (4.50)  (n=939) |

**Risk adjustment as exposure variable**

Supplementary Table 5. Cross-sectional associations between risk adjustment (continuous exposure) and emotional symptoms at age 11 (continuous outcome), complete case sample (n=10,396).

| Sample overall | SDQ change for a 1-point increase in risk adjustment | P value |
| --- | --- | --- |
| Model 1a. Univariable (n=10,396) | -.13 (-.19 to -.08) | <.0001 |
| Model 1b. Model 1a adjusted^a^ | -.04 (-.09 to .01) | .121 |
| Model 1c. Model 1b adjusted for gender | -.04 (-.09 to .01) | .124 |
| Sub-group analyses by gender | SDQ change for a 1-point increase in risk adjustment | P value |
| Model 2a. Univariable, females (n= 5159) | -.14 (-.21 to -.07) | <.0001 |
| Model 2b. Model 2a adjusted^b^ | -.05 (-.13 to .02) | .128 |
|  |  |  |
| Model 3a. Univariable, males (n=5237) | -.13 (-.18 to -.07) | <.0001 |
| Model 3b. Model 3a adjusted^b^ | -.03 (-.09 to .04) | .436 |

^a^Adjusted for confounders measured at or as close as possible to the time of the exposure: family income, maternal education, child age, child ethnicity, child IQ, main carer depressive symptoms.

^b^Adjusted for the above confounders and, in addition, stage of breast development in females or stage of facial hair development in males.

Supplementary Table 6. Cross-sectional associations between risk adjustment (continuous exposure) and depressive symptoms at age 14 (continuous outcome), complete case sample (n=8628).

| Sample overall | MFQ change for a 1-point increase in risk adjustment | P value |
| --- | --- | --- |
| Model 1a. Univariable (n=8628) | -.17 (-.33 to -.02) | .029 |
| Model 1b. Model 1a adjusted^a^ | -.10 (-.26 to .06) | .205 |
| Model 1c. Model 1a adjusted for sex | .03 (-.13 to .19) | .699 |
| Sub-group analyses by gender | MFQ change for a 1-point increase in risk adjustment | P value |
| Model 2a. Univariable, females (n=4354) | -.07 (-.33 to .19) | .588 |
| Model 2b. Model 2a adjusted^b^ | .05 (-.22 to .32) | .714 |
|  |  |  |
| Model 3a. Univariable males (n=4274) | -.01 (-.18 to .15) | .883 |
| Model 3b. Model 3a adjusted^b^ | .01 (-.16 to .18) | .905 |

^a^Adjusted for confounders measured at or as close as possible to the time of the exposure: family income, maternal education, child age, child ethnicity, child IQ, main carer depressive symptoms.

^b^ Adjusted for the above confounders and, in addition, stage of breast development in females or stage of facial hair development in males.

Supplementary Table 7. Longitudinal association between risk adjustment (continuous exposure) at age 11 and depressive symptoms (continuous outcome) at age 14, complete cases (n=8418).

| Sample overall | MFQ change for a 1-point increase in risk adjustment | P value |
| --- | --- | --- |
| Model 1a. Univariable (n= 8,418) | -.03 (-.18 to .12) | .666 |
| Model 1b. Model 1a adjusted^a^ | .09 (-.07 to .25) | .275 |
| Model 1c. Model 1b adjusted for sex | .12 (-.03 to .28) | .123 |
| Sub-group analyses by gender | MFQ change for a 1-point increase in risk adjustment | P value |
| Model 2a. Univariable, females (n=4257) | -.03 (-.30 to .24) | .810 |
| Model 2b. Model 2a adjusted^b^ | .07 (-.22 to .35) | .635 |
|  |  |  |
| Model 3a. Univariable, males (n=4161) | .01 (-.15 to .18) | .858 |
| Model 3b. Model 3a adjusted^b^ | .15 (-.01 to .32) | .069 |

^a^Adjusted for confounders measured at or as close as possible to the time of the exposure: family income, maternal education, main carer depressive symptoms, child age, child ethnicity, child IQ and SDQ total difficulties score.

^c^Adjusted for the above confounders and, in addition, stage of breast development in females or stage of facial hair development in males.

**Imputed associations with risk taking**

Supplementary Table 8. Cross-sectional associations between risk taking (continuous exposure) and emotional symptoms at age 11 (continuous outcome), multiply imputed sample (n=12,355).

| Sample overall | SDQ change for a 20-point increase in risk taking | P value |
| --- | --- | --- |
| Model 1 Univariable (n=12,355) | -.01 (-.07 to .04) | .617 |
| Model 2: Model 1 adjusted^a^ | -.03 (-.08 to .02) | .221 |
| Model 3: Model 2 adjusted for sex | -.03 (-.08 to .03) | .320 |
| Sub-group analyses by gender | SDQ change for a 20-point increase in risk taking | P value |
| Model 4: Univariable, females (n=6144) | .02 (-.05 to .10) | .522 |
| Model 5: Model 4 adjusted^b^ | -.03 (-.09 to .04) | .445 |
|  |  |  |
| Model 6: Univariable males (n=6211) | .02 (-.06 to .09) | .646 |
| Model 7: Model 6 adjusted^b^ | -.03 (-.10 to .05) | .481 |

^a^Adjusted for confounders measured at or as close as possible to the time of the exposure: family income, maternal education, child age, child ethnicity, child IQ, main carer depressive symptoms.

^b^Adjusted for the above confounders and, in addition, stage of breast development in females and stage of facial hair development in males.

Supplementary Table 9. Cross-sectional associations between risk taking (continuous exposure) and depressive symptoms at age 14 (continuous outcome), imputed sample (n=10,578).

| Sample overall | MFQ change for a 20-point increase in risk taking | P value |
| --- | --- | --- |
| Model 1 Univariable (n=10,578) | -.58 (-.75 to -.41) | <.0001 |
| Model 2: Model 1 adjusted^a^ | -.44 (-.62 to -.26) | <.0001 |
| Model 3: Model 2 adjusted for sex | -.01 (-.19 to .17) | .928 |
| Sub-group analyses by gender | MFQ change for a 20-point increase in risk taking | P value |
| Model 4: Univariable, females (n=5324) | -.05 (-.38 to .28) | .776 |
| Model 5: Model 4 adjusted^b^ | -.08 (-.41 to .24) | .605 |
|  |  |  |
| Model 6: Univariable males (n=5254) | .05 (-.17 to .26) | .649 |
| Model 7: Model 6 adjusted^b^ | .05 (-.17 to .26) | .679 |

^a^Adjusted for confounders measured at or as close as possible to the time of the exposure: family income, maternal education, child age, child ethnicity, child IQ, main carer depressive symptoms.

^b^ Adjusted for the above confounders and, in addition, stage of breast development in females and stage of facial hair development in males.

Supplementary Table 10. Longitudinal association between risk taking (continuous exposure variable) at age 11 and depressive symptoms (continuous outcome) at age 14, imputed sample (n=12,355).

| Sample overall | MFQ change for a 20-point increase in risk taking | P value |
| --- | --- | --- |
| Model 1 Univariable (n=12,355). | -.60 (-.76 to -.43) | <.0001 |
| Model 2: Model 1 adjusted^a^ | -.30 (-.46 to -.14) | <.0001 |
| Model 3: Model 2 adjusted for sex | -.13 (-.29 to .03) | .112 |
| Sub-group analyses by gender | MFQ change for a 20-point increase in risk taking | P value |
| Model 4: Univariable, females (n=6144) | -.21 (-.44 to .03) | .089 |
| Model 5: Model 4 adjusted^b^ | -.24 (-.47 to -.00) | .047 |
|  |  |  |
| Model 6: Univariable males (n=6211) | .06 (-.15 to .26) | .583 |
| Model 7: Model 6 adjusted^b^ | -.01 (-.20 to .19) | .936 |

^a^Adjusted for confounders measured at or as close as possible to the time of the exposure: family income, maternal education, main carer depressive symptoms, child age, child ethnicity, child IQ and SDQ total difficulties score.

^c^Adjusted for the above confounders and, in addition, stage of breast development in females and stage of facial hair development in males.

**References**

Flouri, E., & Ioakeimidi, S. (2018). Maternal depressive symptoms in childhood and risky behaviours in early adolescence. *European Child & Adolescent Psychiatry*, *27*, 301–308. https://doi.org/10.1007/s00787-017-1043-6

Goodman, A., & Goodman, R. (2009). Strengths and difficulties questionnaire as a dimensional measure of child mental health. *Journal of the American Academy of Child and Adolescent Psychiatry*, *48*(4), 400–403. https://doi.org/10.1097/CHI.0b013e3181985068

Joinson, C., Heron, J., Araya, R., Paus, T., Croudace, T., Rubin, C., … Lewis, G. (2012). Association between pubertal development and depressive symptoms in girls from a UK cohort. *Psychological Medicine*, *42*(12), 2579–2589. https://doi.org/10.1017/S003329171200061X

Kelly, Y., Zilanawala, A., Sacker, A., Hiatt, R., & Viner, R. (2017). Early puberty in 11-year-old girls: Millennium Cohort Study findings. *Archives of Disease in Childhood*, *102*(3), 232–237. https://doi.org/10.1136/archdischild-2016-310475

Kessler, R. C., Andrews, G., Colpe, L. J., Hiripi, E., Mroczek, D. K., Normand, S. L. T., … Zaslavsky, A. M. (2002). Short screening scales to monitor population prevalences and trends in non-specific psychological distress. *Psychological Medicine*, *32*(6), 959–976.

Lewis, G., Ioannidis, K., van Harmelen, A.-L., Neufeld, S., Stochl, J., Lewis, G., … Goodyer, I. (2018). The association between pubertal status and depressive symptoms and diagnoses in adolescent females: A population-based cohort study. *PLOS ONE*, *13*(6), e0198804. Retrieved from http://www.ncbi.nlm.nih.gov/pubmed/29912985

Petersen, A. C., Crockett, L., Richards, M., & Boxer, A. (1988). A self-report measure of pubertal status: Reliability, validity, and initial norms. *Journal of Youth and Adolescence*, *17*(2), 117–133. https://doi.org/10.1007/BF01537962
