# Supplementary material for: Risk-taking to obtain reward: sex differences and associations with emotional and depressive symptoms in a nationally representative cohort of UK adolescents
Source: Psychol Med. 2021 Jan 12;52(13):2805–13. doi: 10.1017/S0033291720005000 (PMC9647510; doi:10.1017/S0033291720005000)
Supplement: Supplementary file 1 [file S0033291720005000sup.zip › S0033291720005000sup002.docx]

5531 lost by age 11 follow-up

13,287 lost by age 14 follow-up

18,818 children recruited for MCS cohort at birth

13,287 took part in MCS at age 11

12,355 did CGT at age 11

11,714 took part in MCS at age 14

11,931 did SDQ at age 11

10,396 had data on all confounders

8628 had data on all confounders

10,578 did CGT at age 14

10,246 did MFQ at age 14

8418 of these had MFQ at age 14

10,388 had the total SDQ score at baseline, for adjustment in longitudinal analysis

932 did not do CGT at age 11

932 did not do CGT at age 14

424 did not do SDQ at age 11

1535 with incomplete data confounders

332 did not do SDQ at age 11

1618 with incomplete data confounders

Figure 1. Flow of participants through the study
