# Supplementary figures and images for: Risk-taking to obtain reward: sex differences and associations with emotional and depressive symptoms in a nationally representative cohort of UK adolescents
Source: Psychol Med. 2021 Jan 12;52(13):2805–13. doi: 10.1017/S0033291720005000 (PMC9647510; doi:10.1017/S0033291720005000)

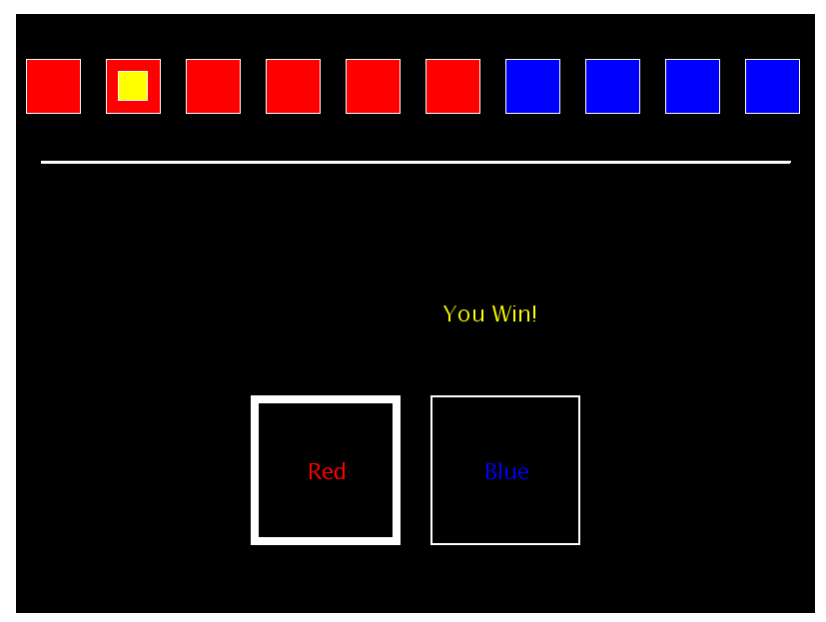


Supplementary Figure 2. The betting-stage of the Cambridge Gambling Task with a red:blue ratio of 6:4.

Supplement: Supplementary file 1 [file S0033291720005000sup.zip › S0033291720005000sup003.docx]
